# Association Between Glycemic Parameters and Severity of Periodontitis in Nepal: A Hospital‐Based Cross‐Sectional Study

**DOI:** 10.1155/ijod/3153277

**Published:** 2026-08-07

**Authors:** Shristi Kafle, Abhishek Gupta, Saurav Khatiwada

**Affiliations:** ^1^ Department of Periodontology and Oral Implantology, Chitwan Medical College, Bharatpur 44207, Nepal, cmc.edu.np; ^2^ Department of Oral Medicine and Radiology, Chitwan Medical College, Bharatpur 44207, Nepal, cmc.edu.np; ^3^ Department of Endocrinology, Chitwan Medical College, Bharatpur 44207, Nepal, cmc.edu.np

**Keywords:** diabetes mellitus, glycated hemoglobin (HbA1c), glycemic control, Nepal, periodontitis

## Abstract

**Background:**

Periodontitis is a prevalent inflammatory disease affecting the supporting structures of the teeth and has been strongly associated with systemic conditions such as diabetes mellitus. Poor glycemic control has been reported to exacerbate periodontal tissue destruction; however, evidence from many developing countries remains limited. This study aimed to evaluate the association between glycemic parameters and periodontitis severity in a hospital‐based population in Nepal.

**Methods:**

A hospital‐based cross‐sectional study was conducted among 396 adult patients attending the dental outpatient department of a tertiary care hospital in Nepal. Participants were categorized according to the severity into mild (*n* = 132), moderate (*n* = 132), and severe (*n* = 132) groups based on periodontal clinical examination. The terminology was aligned with the 2017 World Workshop classification of periodontal and peri‐implant diseases and conditions. Full periodontal grading was not assigned because longitudinal data on disease progression were unavailable. Patients presenting with a molar‐incisor pattern suggestive of the previously described aggressive periodontitis phenotype were excluded. Demographic variables, smoking status, periodontal clinical parameters, and glycemic indicators, including fasting blood glucose (FBG), postprandial blood sugar (PPBS), and glycated hemoglobin (HbA1c), were recorded. Descriptive statistics were used to summarize the study variables. Differences in glycemic parameters across periodontitis severity groups were assessed using the Kruskal–Wallis test. Spearman correlation analysis was applied to assess the association between glycemic markers and periodontal clinical parameters. Ordinal logistic regression was conducted to determine the independent predictors of increasing severity of periodontitis.

**Results:**

Glycemic parameters worsened with increasing severity of periodontitis. Patients with severe periodontitis had markedly increased levels of FBG, PPBS, and HbA1c in comparison to those with mild and moderate periodontitis (*p*  < 0.001). Periodontal clinical parameters, such as probing depth and clinical attachment loss (CAL), were most strongly correlated with HbA1c (*p*  < 0.001). Ordinal logistic regression analysis revealed that HbA1c was the most significant independent predictor of increasing severity of periodontitis, followed by age and current smoking status.

**Conclusions:**

Severe periodontitis was significantly associated with worsening glycemic status. Of all the assessed glycemic markers, HbA1c exhibited the most robust relationship with periodontal destruction. These findings highlight the importance of glycemic control in periodontal health and support the need for integrated medical–dental management strategies for patients with metabolic disorders.

## 1. Introduction

Periodontal disease includes a group of diseases that lead to inflammatory destruction of the supportive structures of teeth and are among the most common oral health conditions globally [1, 2]. Periodontitis is characterized by progressive destruction of the periodontal ligament and the alveolar bone, which may result in tooth mobility and tooth loss if left untreated. Periodontal disease results from a complex interaction between pathogenic microorganisms within the dental biofilm and the host immune‐inflammatory response, which determines the degree of periodontal tissue destruction [1–3].

The classification of periodontal diseases has evolved over time. The 2017 World Workshop classification replaced the previous distinction between “chronic” and “aggressive” periodontitis with a unified diagnosis of periodontitis, further characterized by stage and grade according to severity, complexity, extent, risk factors, and anticipated rate of progression [4–6]. Therefore, in the present manuscript, the term “periodontitis” is used instead of “chronic periodontitis” to align with the current classification framework. Although this study categorized participants analytically into mild, moderate, and severe periodontitis groups based on clinical periodontal destruction, the findings are interpreted in relation to the contemporary staging framework.

Numerous systemic conditions have been shown to affect the host response and alter periodontal disease progression, one of which is diabetes mellitus, which has received considerable attention in this area [4–6]. Diabetes mellitus is a metabolic disorder with chronic hyperglycemia due to defects in insulin secretion, insulin action, or both. Chronic hyperglycemia is implicated in end‐organ damage and disease progression and has been shown to negatively influence oral health status.

Several epidemiological and clinical studies have shown a significant association between diabetes and periodontal disease, with periodontitis historically described as the sixth complication of diabetes [7–9].

Patients with poor glycemic control show deeper periodontal pockets, increased clinical attachment loss (CAL), and higher rates of tooth loss compared with well‐controlled patients [7, 10–12]. Population‐based studies have also shown that periodontitis is more prevelant and more severe among hyperglycemic individuals compared with nondiabetics [13, 14].

Fasting blood glucose (FBG), postprandial blood sugar (PPBS), and glycated hemoglobin (HbA1c) are the commonly used glycemic parameters to monitor metabolic control in diabetes. Among these markers, HbA1c reflects average blood glucose levels during the preceding two to 3 months and may be regarded as a valid indicator of long‐term glycemic status. Studies have found that higher HbA1c levels are associated with greater periodontal destruction, such as deeper probing depth and increased CAL [15]. In adults with diabetes, increased periodontal inflammatory load as quantified by the periodontal inflamed surface area has been found to correlate with poor glycemic control and risk for diabetes‐related morbidity [15, 16]. Similar associations have been observed between glycemic status and periodontitis severity in both type 1 [17] and type 2 [18, 19] diabetic populations.

The relationship between diabetes and periodontitis is bidirectional and well‐recognized, as hyperglycemia may amplify the host inflammatory response to periodontal infection, thereby accelerating periodontal tissue destruction. Multiple biological mechanisms have been hypothesized to account for the association between hyperglycemia and the destruction of periodontal tissues. Long‐standing hyperglycemia increases advanced glycation end‐products (AGEs) and oxidative stress, which could worsen the inflammatory reaction in oral tissues. Elevated levels of inflammatory mediators such as interleukins (ILs) and tumor necrosis factor (TNF) have been found to be higher in patients with both diabetes mellitus and periodontitis, implying that poor glycemic control can enhance periodontal inflammation, leading to tissue destruction [20, 21]. Moreover, apart from having persistent chronic hyperglycemia, some studies recently reported that glucose variability might be a factor contributing to periodontal inflammation and involved in the severity of the diseases [1].

In the reverse direction, periodontal inflammation may negatively affect metabolic control through systemic inflammatory pathways [4]. Follow‐up studies [22] demonstrated that glycemic status can predict periodontal disease progression over time. Conversely, multiple studies, including systematic reviews, report that glycemic control is modestly improved with periodontal treatment in patients with diabetes [23]. These findings highlight the importance of looking at periodontal health as an essential component of comprehensive care for metabolic diseases.

While there is a growing body of international evidence connecting diabetes with periodontal disease, little data exist from many low‐ and middle‐income countries. Diabetes mellitus and periodontal disease are important public health problems in Nepal, where both are increasingly prevalent, but limited studies have reported the association between them among the clinical population. In this respect, knowledge of the association between glycemic parameters and severity of periodontitis in this population might improve interprofessional strategies in patient care.

Thus, the current study was conducted to evaluate the association between glycemic parameters and periodontitis severity in a hospital‐based dental setting in Nepal. The aim of the study was specifically to assess HBA1c levels, fasting blood sugar, and PPBS in relation to clinical markers of periodontal destruction. This cross‐sectional study aims to provide local evidence by examining these associations within a hospital‐based population and may support integrated periodontal disease management and systemic metabolic health care.

## 2. Materials and Methods

### 2.1. Study Design and Setting

This hospital‐based cross‐sectional study was carried out with the objective of assessing the relationship between glycemic parameters and periodontitis severity among adult patients visiting the dental outpatient department of a tertiary care teaching hospital in Nepal from July 2024 to December 2025. A cross‐sectional design was chosen to assess the relationship between periodontal clinical parameters and glycemic status in a defined clinical population at a single point in time.

### 2.2. Study Population

The study participants included adult patients attending the dental outpatient department during the study period and diagnosed with periodontitis based on the clinical periodontal examination. Periodontitis was diagnosed according to the 2017 World Workshop Classification of periodontal and peri‐implant diseases and conditions. A diagnosis of periodontitis was established when interdental CAL was detectable at two or more non‐adjacent teeth, or when buccal/oral CAL of ≥ 3 mm with probing depth (PD) > 3 mm was present at two or more teeth, provided that the attachment loss could not be attributed to non‐periodontal causes [24–28]. In accordance with the current classification system, the term “periodontitis” was used instead of “chronic periodontitis.” Participants were categorized into mild, moderate, and severe periodontitis groups for analytical purposes based on the extent of periodontal tissue destruction assessed by probing depth and CAL. These categories were interpreted in relation to the contemporary staging framework, where disease severity and complexity are primarily reflected by CAL, probing depth, radiographic bone loss, and tooth loss due to periodontitis. All consecutive patients who fulfilled the eligibility criteria were screened and recruited after written informed consent.

### 2.3. Inclusion Criteria

Participants were eligible if they met the following criteria:

1. People aged 18 years and older.
2. A case of periodontitis diagnosed through clinical periodontal examination according to the 2017 World Workshop classification framework.
3. Having a minimum of 20 natural teeth.
4. Laboratory values for FBG, PPBS, and glycated hemoglobin (HbA1c).
5. Patients with type 2 diabetes mellitus or altered glycemic parameters were included. Diabetes status was confirmed using medical history, available medical records, current use of antidiabetic medication, and/or biochemical criteria based on recognized diagnostic thresholds.

### 2.4. Exclusion Criteria

Individuals were excluded based on the presence of any of the following:

1. History of periodontal treatment in the last 6 months.
2. Receipt of systemic antibiotics in the past 3 months.
3. Other systemic condition(s) (other than diabetes mellitus itself) that may affect periodontal status.
4. Pregnancy or lactation.
5. Those who refused to give written consent.
6. Patients with type 1 diabetes mellitus were excluded because type 1 and type 2 diabetes mellitus have distinct pathophysiological characteristics, age of onset, clinical course, and metabolic profiles, which may influence the periodontal status differently.
7. Patients presenting with a molar‐incisor pattern suggestive of the previously described aggressive periodontitis phenotype were excluded to maintain a clinically homogeneous study population.

### 2.5. Sample Size and Sampling Technique

One‐way ANOVA (fixed effects model) was employed to estimate sample sizes for comparisons of glycemic parameters across three groups corresponding to different periodontitis severity. It followed the conventional ANOVA formulation:

N=Z12−α/+Z1−β/f2,

where *f* is Cohen’s effect size for ANOVA defined as:

f=σm/σ,

where *σm* is the standard deviation of group means and *σ* is the within‐group standard deviation. With *α* = 0.05, statistical power (1−*β*) set at 0.90, and moderate effect size (*f* = 0.25), compatible with previous studies that assessed periodontal–glycemic associations [29, 30], the overall minimum sample size necessary for a cohort of 252 subjects was calculated using 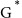
Power (*F*‐test: fixed effects, omnibus ANOVA).

For the purposes of obtaining adequate statistical power, external validity, and balancing of groups, the sample size was increased to 360 participants (120 per group). Based on a 10% estimated nonresponse or incompletion rate, the final sample size was *n* = 396 based on equal allocation of cases for mild (*n* = 132), moderate (*n* = 132), and severe periodontitis groups (*n* = 132), enabling maximization of both statistical efficiency and comparability.

### 2.6. Data Collection

Data were gathered from the clinical periodontal examination and laboratory records review. Demographic, behavioral, clinical, and biochemical variables were recorded in a structured data collection form.

The following variables were recorded:

• Age.
• Gender.
• Body mass index (BMI).
• Smoking status (current smoker, former smoker, and nonsmoker).
• FBG (mg/dL).
• PPBS (mg/dL).
• Glycated hemoglobin (HbA1c, %).
• Probing depth (mm) CAL (mm).
• Bleeding on probing.

### 2.7. Periodontal Examination

A trained examiner performed the clinical periodontal examination using a calibrated periodontal probe under standardized clinical conditions. Probing depth was measured at six sites per tooth, and the final report for each participant represented the average of probing depth (mm). The extent of periodontal tissue destruction was assessed by CAL.

Periodontitis was diagnosed and categorized using clinical periodontal parameters, including probing depth, CAL, and bleeding on probing. The periodontal diagnostic approach was based on the 2017 World Workshop classification of periodontal and peri‐implant diseases and conditions [24–28]. For analytical comparison, participants were divided into three severity groups according to increasing periodontal tissue destruction:

i. Mild periodontitis
ii. Moderate periodontitis
iii. Severe periodontitis

These categories reflected increasing levels of periodontal tissue destruction observed during the clinical examination. Although full staging and grading were not assigned because longitudinal evidence of disease progression and complete radiographic staging data were not available for all participants, severity grouping was based on CAL and probing depth and interpreted in relation to the contemporary staging framework.

### 2.8. Glycemic Parameters

Three biochemical markers were used to assess the glycemic status are as follows:

i. FBG: in mg/dL after an overnight fast.
ii. PPBS: sugar measured 2 h after eating.
iii. Glycated hemoglobin (HbA1c): a percentage that reflects the average blood glucose level for the prior two to 3 months.

Glycemic laboratory data were obtained from patient electronic medical records or performed as part of routine clinical investigations. Diabetes mellitus was diagnosed or confirmed based on documented medical history, use of antidiabetic medication, and/or laboratory criteria consistent with recognized diagnostic recommendations. According to the American Diabetes Association criteria, diabetes may be diagnosed using fasting plasma glucose ≥126 mg/dL, 2‐h plasma glucose ≥200 mg/dL during an oral glucose tolerance test, HbA1c ≥6.5%, or random plasma glucose ≥200 mg/dL in the presence of classic symptoms of hyperglycemia [28, 29]. In the present study, only patients with type 2 diabetes mellitus or altered glycemic parameters were included, while patients with type 1 diabetes mellitus were excluded.

### 2.9. Data Management

Data collected were entered into a structured spreadsheet on Microsoft Excel (Microsoft 365, Microsoft Corporation, Redmond, WA, USA) and subsequently imported into statistical software for analysis. The dataset was checked for completeness, consistency, and entry errors before conducting any analysis. Normality and outliers were also assessed for continuous variables.

### 2.10. Statistical Analysis

IBM SPSS Statistics Version 26.0 (IBM Corp., Armonk, NY, USA) was used to analyze the data. Demographic data, periodontal clinical findings, and glycemic measurements were summarized using descriptive statistics. Continuous variables are expressed as mean ± standard deviation and categorical variables as frequencies and percentages.

The Kruskal–Wallis test was used for the analysis of differences in glycemic parameters between three degrees of periodontitis severity. For categorical variables (e.g., gender and smoking status), the association with periodontitis severity was tested using the chi‐square test.

This analysis explored the association between glycemic measures and periodontal clinical parameters, such as probing depth and CAL, by using Spearman correlation analysis.

Ordinal logistic regression analysis was performed to assess independent factors associated with the increasing severity of periodontitis. Adjusted odds ratios (OR) with 95% confidence intervals (CI) were derived to assess the strength of these associations after controlling for possible confounding covariates. Statistical significance was defined as *p*  < 0.05.

### 2.11. Ethical Considerations

Ethical approval was granted by the Institutional Review Committee of the respective institution before study commencement (IRC Ref Number: CMC‐IRC/080/081‐150). All participants were briefed regarding the objectives and methods of the study, and written informed consent was secured before their entry into the study. Confidentiality of our participants was strictly upheld, and all data were anonymized prior to analysis.

## 3. Results

**Figure 1:**
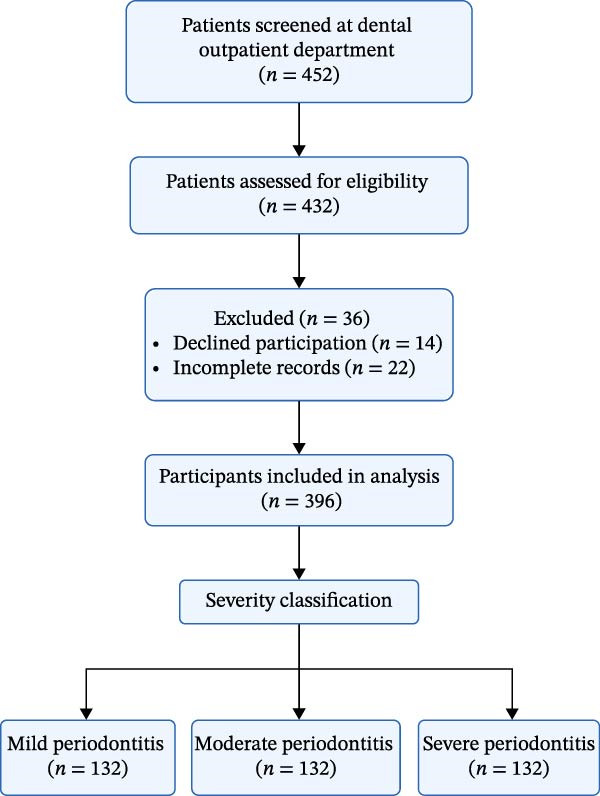
Flow diagram of participant screening, eligibility assessment, and inclusion in the study.

A total of 396 participants were included in the analysis and categorized according to the severity of chronic periodontitis into mild (*n* = 132), moderate (*n* = 132), and severe (*n* = 132) groups. The process of participant screening, eligibility assessment, and inclusion in the study is illustrated in Figure 1.

### 3.1. Demographic and Clinical Characteristics of the Study Population

The demographic and behavioral characteristics of the study participants are presented in Table 1. The mean age increased progressively with increasing severity of periodontitis, with participants in the severe group being older than those in the mild and moderate groups (*p*  < 0.001). Post hoc intergroup comparison showed that age differed significantly between all three groups. BMI also showed a modest increase across severity categories (*p* = 0.041). Post hoc analysis indicated that BMI was significantly higher in the severe group than in the mild group, whereas the mild versus moderate and moderate versus severe comparisons were not statistically significant.

**Table 1:** Demographic and behavioral characteristics of participants according to severity of chronic periodontitis.

Variable	Mild (*n* = 132)	Moderate (*n* = 132)	Severe (*n* = 132)	*p*‐Value
Age (years) mean ± SD	49.6 ± 10.2	55.1 ± 9.8	62.7 ± 11.1	<0.001
Gender, *n* (%)				0.18
Male	64 (48.5)	69 (52.3)	78 (59.1)	—
Female	68 (51.5)	63 (47.7)	54 (40.9)	—
BMI (kg/m²), mean ± SD	24.8 ± 3.9	25.9 ± 4.0	26.6 ± 4.5	0.041
Smoking status, *n* (%)				0.003
Nonsmoker	74 (56.1)	61 (46.2)	46 (34.8)	—
Former smoker	29 (22.0)	32 (24.2)	31 (23.5)	—
Current smoker	29 (22.0)	39 (29.5)	55 (41.7)	—

*Note:* Continuous variables are presented as mean ± SD; categorical variables as *n* (%). *p*‐Values were calculated using the Kruskal–Wallis test for continuous variables and the chi‐square test for categorical variables.

With respect to smoking status, the proportion of current smokers was higher in the severe periodontitis group compared with the mild and moderate groups (*p* = 0.003). Intergroup comparison showed that current smoking was significantly more frequent in the severe group than in the mild group.

### 3.2. Glycemic Parameters According to Periodontitis Severity

The distribution of glycemic parameters according to the severity of periodontitis is shown in Table 2. Mean FBG, PPBS, and HbA1c levels increased progressively from mild to severe periodontitis. Participants with severe periodontitis exhibited the highest glycemic values across all parameters. Differences between the groups were statistically significant for all glycemic indicators (*p*  < 0.001). Post hoc intergroup comparisons showed that FBG, PPBS, and HbA1c differed significantly between all three severity groups.

**Table 2:** Glycemic parameters according to severity of chronic periodontitis.

Parameter	Mild (*n* = 132)	Moderate (*n* = 132)	Severe (*n* = 132)	*p*‐Value
Fasting blood glucose (mg/dL)	94.2 ± 6.8	111.5 ± 9.4	146.8 ± 28.3	<0.001
Postprandial blood sugar (mg/dL)	128.6 ± 12.1	168.7 ± 19.4	246.2 ± 55.6	<0.001
HbA1c (%)	5.5 ± 0.4	6.6 ± 0.5	9.8 ± 1.7	<0.001

*Note:* Values are presented as mean ± SD. *p*‐Values were calculated using the Kruskal–Wallis test.

### 3.3. Periodontal Clinical Parameters

The distribution of periodontal clinical parameters across severity groups is presented in Table 3. Mean probing depth and CAL increased progressively from mild to severe periodontitis. Participants in the severe group demonstrated the highest mean probing depth and CAL values. Post hoc analysis showed significant differences in probing depth and CAL between all three severity groups. Bleeding on probing was also more frequently observed in participants with moderate and severe forms of periodontitis. Pairwise comparison showed that bleeding on probing was significantly different across all three groups.

**Table 3:** Periodontal clinical parameters according to severity of chronic periodontitis.

Parameter	Mild (*n* = 132)	Moderate (*n* = 132)	Severe (*n* = 132)	*p*‐Value
Probing depth (mm)	2.9 ± 0.3	4.2 ± 0.5	6.3 ± 0.8	<0.001
Clinical attachment loss (mm)	3.1 ± 0.4	4.8 ± 0.7	6.9 ± 1.1	<0.001
Bleeding on probing *n* (%)	38 (28.8)	69 (52.3)	101 (76.5)	<0.001

*Note:* Continuous variables are presented as mean ± SD; categorical variables are presented as *n* (%). *p*‐Values were calculated using the Kruskal–Wallis test for continuous variables and the chi‐square test for bleeding on probing.

### 3.4. Correlation Between Glycemic Parameters and Periodontal Destruction

Spearman correlation analysis between glycemic parameters and periodontal clinical measures is presented in Table 4. Positive correlations were observed between glycemic indicators and both probing depth and CAL. HbA1c demonstrated the strongest correlation with periodontal parameters. Since Table 4 presents correlation coefficients rather than group comparisons, intergroup superscript letters were not applicable.

**Table 4:** Spearman correlation between glycemic parameters and periodontal clinical measures.

Variable	Probing depth (*r*)	Clinical attachment loss (*r*)	*p*‐Value
HbA1c (%)	0.63	0.67	<0.001
Fasting blood glucose	0.54	0.57	<0.001
Postprandial blood sugar	0.59	0.61	<0.001

*Note:* Spearman correlation coefficients are shown. All correlations were statistically significant at *p* < 0.001.

### 3.5. Predictors of Increasing Periodontitis Severity

Ordinal logistic regression analysis was performed to identify predictors of increasing severity of chronic periodontitis. The results are shown in Table 5. HbA1c remained the strongest independent predictor of periodontitis severity. Age and current smoking status were also significantly associated with higher disease severity, whereas BMI was not statistically significant in the adjusted model.

**Table 5:** Ordinal logistic regression analysis predicting severity of chronic periodontitis.

Predictor	Adjusted OR	95% CI	*p*‐Value
HbA1c (%)	1.91	1.55–2.34	<0.001
Fasting blood glucose	1.02	1.01–1.03	0.002
Postprandial blood sugar	1.01	1.00–1.02	0.015
Age	1.03	1.01–1.05	0.004
BMI	1.04	0.99–1.09	0.083
Current smoking	1.72	1.18–2.51	0.005

## 4. Discussion

The present hospital‐based cross‐sectional study investigated the association between glycemic parameters and the severity of periodontitis among adult patients attending a tertiary care hospital in Nepal. The findings of this study demonstrated a clear and statistically significant direct relationship between worsening glycemic status and the increasing severity of periodontitis. Participants with severe periodontitis exhibited substantially higher FBG, PPBS, and HbA1c levels compared with individuals with mild and moderate periodontitis. In addition, HbA1c showed the strongest association with periodontal clinical parameters and emerged as the most significant independent predictor of increasing disease severity in the regression model. Overall, these findings suggest a strong association between worsening metabolic control and increased periodontal tissue destruction in this patient population.

The interpretation of these findings should be considered in light of the clarified periodontal and diabetes classifications used in this study. In accordance with the current 2017 World Workshop classification, the term “periodontitis” was used instead of the previously used term “chronic periodontitis.” Although participants were categorized into mild, moderate, and severe groups for analytical comparison, these categories were based on increasing clinical periodontal destruction assessed using probing depth and CAL and were interpreted in relation to the contemporary staging framework. Full periodontal grading was not assigned because longitudinal information on disease progression and complete grading‐related data were not available for all participants. This point is particularly relevant because diabetes is recognized as an important modifier of periodontitis grading. Therefore, glycemic status was incorporated analytically through FBG, PPBS, and HbA1c rather than being used to assign formal periodontal grades. Furthermore, the present study focused on adult patients with type 2 diabetes mellitus or altered glycemic parameters, while patients with type 1 diabetes mellitus were excluded. This distinction is important because type 1 and type 2 diabetes mellitus differ in pathophysiology, age of onset, clinical course, and metabolic profile, which may influence periodontal outcomes differently. Consequently, the findings should be generalized primarily to adult patients with type 2 diabetes mellitus or dysglycemia in similar hospital‐based clinical settings.

These results agree with the literature, where poor glycemic control correlates with increased periodontal destruction [6, 7]. Studies have demonstrated that subjects with uncontrolled diabetes have deeper periodontal pockets, more CAL, and a higher risk of tooth loss than those with well‐controlled glycemic status [29, 30]. In addition, population‐based epidemiologic studies have shown a high correlation between higher levels of HbA1c and the prevalence/severity of periodontitis in adults [13, 14]. These findings are in line with the current study report, where progressive deterioration of metabolic control accompanied increased periodontal destruction [15, 16]. Moreover, data from recently conducted clinical and epidemiological studies also strongly support this association as poorer periodontal outcomes correlate significantly with higher HbA1c values [18, 24]. Taken together, these findings indicate that chronic hyperglycemia leads to the dysregulation of the host immune‐inflammatory response, which promotes progression and severity of periodontitis.

The present study found that, among the glycemic parameters assessed, glycated hemoglobin (HbA1c) correlated best with clinical periodontal parameters, including probing depth and CAL, indicating a closer relationship between long‐term glycemic status and periodontitis severity, consistent with the findings of other studies [7, 16, 28]. Glycated hemoglobin (HbA1c) reflects the average concentration of blood glucose over the preceding 2 to 3 months and is considered a reliable biomarker of long‐term glycemic control because it represents cumulative exposure to elevated glucose rather than short‐term fluctuations in blood sugar levels [7, 28–30]. Previous studies suggested that HbA1c correlates more strongly with periodontal inflammatory markers and clinical indices than fasting or postprandial glucose when assessed as an isolated measurement [31–33]. While fasting or single postprandial glucose measurements reflect momentary glycemic status, HbA1c offers a more robust measure of systemic metabolic exposure and is more strongly linked with cumulative periodontal inflammation and clinical indices than isolated glucose readings. Previous observational research has shown that elevated HbA1c levels are associated with increased severity of periodontal parameters such as periodontal inflamed surface area and CAL in individuals with diabetes, suggesting a significant relationship between chronic hyperglycemia and periodontal destruction [34]. Quantitative data further support strong relationships between increased HbA1c levels and significant periodontal health deterioration, such as CAL and periodontal inflammation, indicating that poorer long‐term glycemic control is correlated with more pronounced periodontitis [32]

Studies examining the bidirectional relationship between periodontitis and diabetes have consistently demonstrated that elevated HbA1c levels are associated with increased severity of periodontal destruction, likely due to the effects of chronic hyperglycemia on host immune function and inflammatory responses [7, 8, 10].

Furthermore, recent narrative reviews and syntheses of the literature have identified HbA1c as a key glycemic marker in the context of periodontitis, with elevated HbA1c levels frequently associated with greater severity of periodontal outcomes, likely because chronic hyperglycemia reflects the long‐term metabolic burden contributing to inflammatory and tissue‐destructive processes in the periodontium. A comprehensive 2025 narrative review on the bidirectional relationship between periodontitis and diabetes highlighted elevated HbA1c as a clinical feature that correlates with worsening periodontitis and may be more informative for explaining variation in disease severity than isolated glucose measures [35]. This study confirms that HbA1c is a strong metabolic marker linking glycemic status and severity of periodontitis.

The exact biological basis of this association is still complex but certainly involves many proinflammatory and metabolic pathways. Hyperglycemia induces generation and accumulation of AGEs, which then bind to their cellular receptors (RAGE) expressed on immune cells or periodontal tissue. As a consequence of the increased production of proinflammatory cytokines (TNF‐α, IL‐1β, and IL‐6) due to AGE‐RAGE intracellular signaling cascades, such as nuclear factor‐κB (NF‐κB) systems and various proinflammatory pathways, periodontal inflammation and connective tissue breakdown increase under the effect of AGEs on RAGE [36]. Upregulated glucose can further promote the generation of reactive oxygen species (ROS), which cause oxidative damage to the cell structures and potentiate inflammatory responses [4, 37, 38]. Oxidative stress is known to play a role in aggravating lipid peroxidation, DNA damage, and activated extracellular matrix (ECM)‐degrading matrix metalloproteinases (MMPs) found in periodontal tissues [39]. These mechanisms could result in progressive periodontal attachment loss among diabetics with poor metabolic control. In diabetic patients with periodontitis, higher levels of inflammatory mediators were repeatedly reported in gingival crevicular fluid and periodontal tissues compared to periodontitis subjects without diabetes [40–42], with the exception of studies finding an elevated status particularly when glycemic control was below optimal.

Apart from blood glucose levels, recent literature has demonstrated the association of glycemic variability with periodontal inflammation and disease progression. In parallel, in addition to chronic hyperglycemia, glycemic variability may independently further aggravate the extent of the inflammatory response and periodontal tissue destruction. Greater glucose variability, reflected by higher standard deviation and coefficient of variation, was significantly associated with increased probing depth and CAL in patients presenting for periodontal therapy [43].

Similarly, enhanced glycemic variability has been linked with higher levels of inflammatory cytokines in the gingival crevicular fluid and greater severity of periodontitis. Thus, the authors proposed that both chronic hyperglycemia and glucose variability could be key mediators involved in the pathogenesis of periodontal destruction in subjects with metabolic disorders [44]. Diabetes and periodontal disease are known to be bidirectionally associated. Hyperglycemia is involved in the destruction of periodontal tissues, and periodontal irritation can adversely affect glycemic control too. And systemic inflammatory mediators like IL‐6, TNF‐α and C‐reactive protein can also be increased due to chronic periodontal infection and may potentiate insulin resistance/outweigh metabolic regulation [45]. Longitudinal data indicated that poor glycemic status has preceded future periodontal breakdown (e.g., increased rate of attachment loss progression and increased risk of tooth loss) [7, 9].

There is evidence that periodontal therapy results in a small improvement in glycemic control of patients with diabetes, as evidenced by systematic reviews and randomized clinical interventions [23, 46, 47]. Recent meta‐analytic data have shown that nonsurgical periodontal treatment with scaling and root planing can lower HbA1c values by 0.3%–0.5% within a few months, which suggest the importance of improvements in periodontal health concerning metabolic performance [23].

More recently, longitudinal and interventional studies have also strengthened the findings that periodontal therapy is associated with clinically meaningful reductions in glycated hemoglobin over time. A recent report from a cohort study demonstrated that nonsurgical periodontal therapy was associated with such an improvement in the inflammatory burden of periodontitis, resulting in sustained improvements in HbA1c, emphasizing the systemic effects due to periodontal therapies on glycemic regulation [48]. Recent reports highlight that HbA1c may also play a central mediating role in this bidirectional relationship, as its increased levels aid in the decline of periodontal status and worsening of the disease progression [35]. Such findings further support the incorporation of periodontal care into holistic management approaches for diabetes.

Besides glycemic parameters, this study also revealed age and smoking status to be strong predictors of increasing periodontitis severity. Aging is a known risk factor associated with cumulative periodontal destruction, continuous exposure to microbial biofilm, systemic factors, and environmental risk factors contribute to the progressive loss of periodontal tissue. Hence, the higher mean age among severe disease participants in this study fits with what is well established about the natural history of periodontitis progression [49, 50]. Smoking is another behavioral risk factor for periodontitis, which is known to impair immune responses, reduce vascularity of periodontal tissues, and disrupt normal wound healing processes. Thus, tobacco exposure may contribute to periodontal inflammation and susceptibility to periodontal breakdown [49, 51]. In this study, we found that current smokers were more prevalent among individuals with severe periodontitis, and there is evidence pointing to the role of smoking as an important modifier of periodontitis severity [50].

This study brings significant relationship between glycemic status and periodontitis severity within a Nepalese hospital‐based population. While an essential amount of studies have been done worldwide to investigate the association between diabetes and periodontitis, not much data is available for Nepal and similar South Asian populations. Thus, the results of this study add to the current literature by presenting geographic‐specific clinical evidence that correlates glycemic deterioration with periodontitis progression in this population.

However, a few limitations must be recognized despite the significant results of this study. First, the use of cross‐sectional design did not allow us to conclude causal relationships between glycemic parameters and periodontitis progression. Longitudinal data would be necessary to assess whether poor glycemic control in fact translates into worsening periodontal outcomes over time. Second, this study was performed in a hospital‐based setting, which may limit the generalizability of our findings to the broader community population. The subjects at tertiary dental centers may have more advanced diseases or comorbidities than the general population. Third, some behavioral and lifestyle factors, including oral hygiene habits, dietary habits, and socioeconomic status, were not comprehensively assessed, which may also have an impact on periodontal health. Fourth, although periodontitis severity was categorized using clinical periodontal parameters, full staging and grading according to the 2017 classification could not be assigned because longitudinal progression data and complete grading‐related information were unavailable for all participants. Fifth, because patients with type 1 diabetes mellitus were excluded, the findings may not be generalizable to type 1 diabetes populations.

However, the current study also holds several significant strengths. This is among the largest such studies and included well‐characterized subjects across a spectrum with mild, moderate, and severe periodontitis, which allowed reasonable comparisons by person group.

These encompassed FBG, PPBS, and HbA1c to enable an analysis of metabolic query in multiple glycemic metrics. In addition, standardized periodontal clinical measurements were used to determine the severity of disease, which enhanced the reliability and external applicability of the findings.

The findings of the present study reinforce that metabolic control is relevant for the periodontal status. The correlation between glycemic indicators and periodontitis severity that we found in this study supports the comanagement of diabetes or other metabolic disorders with dental practitioners. Early identification of patients having poor glycemic control, followed by expedited periodontal evaluation, might be an important step in truncating periodontal destruction and deterring deterioration of systemic health. The incorporation of periodontal screening and treatment into routine care for people with diabetes may thus be an important measure to address the coburden of metabolic and oral diseases.

## 5. Clinical Implications

The findings of this study have important implications for clinical practice. Patients with poor glycemic control, particularly those with elevated HbA1c levels, should undergo routine periodontal assessment as they are at increased risk of severe periodontitis. Likewise, dental professionals must be mindful of the potential for referral to glycemic evaluation in patients presenting with severe or rapidly progressing periodontitis.

Integration of periodontal care into diabetes management programmes could help to improve systemic outcomes given the adverse effects of periodontal inflammation on metabolic control. Therefore, early intervention and prevention strategies aimed at both periodontal health and glycemic control may lend to a reduction in the overall disease burden and improved patient outcome.

## 6. Conclusion

The current hospital‐based cross‐sectional study showed a statistically significant association between glycemic parameters and the severity of periodontitis among adult patients in Nepal. Notably, there were significant associations between increased FBG and PPBS and glycated hemoglobin levels, and periodontal tissue destruction. The glycemic parameter with the greatest correlation to periodontal clinical parameters was HbA1c, which emerged as the strongest independent predictor of increasing periodontitis severity. These findings should be interpreted in the context of the periodontal classification approach and diabetes classification used in the study, particularly the inclusion of adults with type 2 diabetes mellitus or altered glycemic status and the exclusion of type 1 diabetes mellitus.

The results add further support to the concept of metabolic dysregulation in periodontitis and the importance of glycemic control in periodontitis. The established correlation further lends support to a global interplay between systemic metabolic status and oral inflammatory disease.

In summary, the findings highlight the role of glycemic status in periodontitis and underscore the need for early diagnosis and monitoring of patients with periodontitis to minimize complications and emphasize interdisciplinary collaboration between dental and medical practices for the management of people with metabolic disorders.

## Author Contributions

All authors contributed to the conception, design, data collection, analysis, and manuscript preparation.

## Funding

The authors received no specific funding for this study.

## Disclosure

All authors have read, agreed, and approved to the publication‐ready version of the manuscript. The authors take full responsibility for the content of the manuscript.

## Ethics Statement

Ethical approval for this study was obtained from the Institutional Review Committee with Reference Number CMC‐IRC/080/081‐150.

## Consent

Written informed consent was obtained from all participants prior to recruitment in the study.

## Conflicts of Interest

The authors declare no conflicts of interest.

## Data Availability

The datasets generated and/or analyzed during the current study are available from the corresponding author upon reasonable request.
